# Timeless: A Large Sample Study on the Temporal Robustness of Affective Responses

**DOI:** 10.3389/fpsyg.2016.00841

**Published:** 2016-06-01

**Authors:** Christopher Postzich, Katarina Blask, Christian Frings, Eva Walther

**Affiliations:** Department of Psychology, University of TrierTrier, Germany

**Keywords:** valence, arousal, duration, large sample study, temporal robustness

## Abstract

Emotion and its effects on other psychological phenomena are frequently studied by presenting emotional pictures for a short amount of time. However, the duration of exposure strongly differs across paradigms. In order to ensure the comparability of affective response elicitation across those paradigms, it is crucial to empirically validate emotional material not only with regard to the affective dimensions valence and arousal, but also with regard to varying presentation times. Despite this operational necessity for the temporal robustness of emotional material, there is only tentative empirical evidence on this issue. To close this gap, we conducted a large sample study testing for the influence of presentation time on affective response elicitation. Two hundred and forty emotional pictures were presented for either 200 or 1000 ms and were rated by 302 participants on the core affect dimensions valence and arousal. The most important finding was that affective response elicitation was comparable for 200 and 1000 ms presentation times, indicating reliable temporal robustness of affective response elicitation within the supra-liminal spectrum. Yet, a more detailed look on the data showed that presentation time impacted particularly on high arousing negative stimuli. However, because these interaction effects were exceedingly small, they must be interpreted with caution and do not endanger the main finding, namely the quite reliable temporal robustness of affective response elicitation. Results are discussed with regard to the comparability of affective response elicitation across varying paradigms.

## Introduction

Emotions and affective reactions are core processes of human behavior. In order to investigate the processing of emotional contents, participants are typically confronted with pictorial material in psychological experiments. Pictures are used because it is presumed that they elicit reliable affective reactions by simultaneously enabling experimental control. Still, there remains a vivid discussion of which dimensions are relevant in emotional processing. The dimensional view of emotion (Wundt, 1896), later supported by Osgood's work (Osgood, 1952), used factor analytic methods (Carroll et al., 1959) and found two main dimensions, termed “valence” and “arousal,” explaining a great deal of variance in affective evaluations. Similarly, Russell (2003) proposed a framework defining the atoms of emotions as core affect, which consists of the two dimensions of valence (pleasant to unpleasant) and arousal (sleepy to activated). These building bricks of emotion classification are often considered to be independent, although there is considerable doubt concerning this relationship of valence and arousal (Kuppens et al., 2013). Yet, there is not only uncertainty on the functional dependencies of affective reactions on a conceptual level, but also on a process level. In particular, the question on the boundary conditions of the process underlying affective response elicitation is still of major importance for the field (cf., Huang et al., 2008). In any case, emotion research strongly relies on the availability of empirically verified affective stimulus material that reliably elicits affective responses across a variety of paradigms targeted at the same processing stages. The necessity of empirically verified affective material in emotion research becomes especially apparent considering the large amount of emotional picture databases. We will now shortly discuss those available databases and highlight the shortcoming to which the conducted large sample study shall respond.

### Existing databases

#### IAPS

The need of emotion research for empirically verified affective material was first addressed by Lang and colleagues who founded the IAPS database (Lang et al., 1993, 2008). Over a period of 10 years, affective pictures were rated by participants on the dimensions of valence, arousal, and dominance. Since then, this database has mainly served two purposes: a better control of the affective content of the stimulus material and accessibility of normed emotional material leading to shared usage by different scientific groups and thereby ensuring a better comparability of studies from different laboratories. The pictures' suitability has also been validated on physiological measures of affect induction and expression (Lang et al., 1993) as well as on neuropsychological methods (Bradley et al., 2007). The IAPS pictures have also proven cross-cultural validity in both an Eastern European (Drace et al., 2013) and a Chilean sample (Dufey et al., 2011).

#### GAPED

Dan-Glauser and Scherer (2011) noted that the IAPS material is prone to habituation effects when presented repeatedly to the same pool of participants, and that new material is therefore needed. They also argued that the semantic content of the pictures was too broad to capture enough specific situations, objects, or emotional categories that some researchers might need (e.g., violation of norms and laws, animal, and human cruelty etc.). To provide new material they installed an own database called the Geneva affective picture database (GAPED), which comprises 730 affective pictures including spiders, snake pictures, and scenes of the violation of legal and moral norms. These pictures were rated on the dimensions of valence, arousal, and the accordance of the stimuli with moral and legal norms.

#### EmoPics and NAPS

Two other databases are the EmoPics database (Wessa et al., 2010) which was developed to supply the IAPS database with new picture material and the Nencki Affective Picture Systems (NAPS; Marchewka et al., 2014). The NAPS database was built in anticipation of the growing demand for empirically validated and normed affective pictures of higher quality and bigger image sizes than provided by the previous databases. The authors also took approach-avoidance motivation into their rating procedure.

While controlling for many stimulus features like picture content, luminance, color, complexity etc., what all the above mentioned databases still lack is control of the pictures' ability to induce affective responses when they are presented only for a short time interval. The present study is exactly concerned with this goal. To the best of our knowledge there has been no research yet that aimed to determine whether short presentation times of stimuli alter their perceived affective content. This appears to be astonishing not only because inducing affect by shortly presented pictures is of crucial importance for a variety of research fields, but also with regard to current theorizing on the processes underlying affective response elicitation. For instance, having a closer look at the attentional and motivational underpinnings of emotion processing quickly unveils the importance of investigating the time course of subjective affective responses. In particular, taking the perspective of a motivated attention account (e.g., Lang et al., 1997; Hamm et al., 2003), the processing of emotional stimuli does not only include a shift in motivational orientation but also a shift in attentional focus. Together these two processes then combine to prepare the organism for an affective response. Against this background it seems plausible to presume that the significance of an emotional event (cf., Bradley, 2009) and in this regard the involvement of the motivational and attentional processes might vary with presentation duration. Unfortunately, the existing databases only provide valence and arousal ratings which were given by participants with no time constraints. It is, however, completely unclear whether these ratings are still valid if participants see those pictures only for a very short period of time.

The current large sample study was designed to overcome this shortcoming. In particular, 240 emotional pictures were presented for either 200 or 1000 ms and were rated by 302 participants on the core affect dimensions valence and arousal. The main objective of the current study is therefore to provide empirically verified data on the temporal robustness of affective response elicitation across paradigms presenting stimuli only for a very short amount of time. To this end, we summarize two groups of different experimental paradigms below, which typically use emotional pictures for eliciting affective responses with either short or long presentation times. So the question is “Are affective responses elicited by emotional pictures in—say the affective priming paradigm—comparable with affective responses in the evaluative conditioning paradigm?”

In the group of emotional paradigms using short stimulus presentation times, evaluative priming procedures like affective priming or affect misattribution are frequently studied. In these paradigms, affective material like pictures or words are often used as shortly presented primes preceding a target object of some class (i.e., a picture, word, Chinese character etc.) and some valence (i.e., positive, negative, or neutral) that has to be classified by some rule (i.e., valence, word, or non-word etc.) while reaction times are recorded (Wentura and Degner, 2010). To clarify the terminology, by priming we refer to short-term sequential priming (Wentura and Rothermund, 2014). Many behavioral studies used the affective priming procedure (e.g., Hermans et al., 1994; Spruyt et al., 2002, 2007; Eder et al., 2012) typically presenting affective primes for 150–200 ms. Additionally, electro-cortical studies have been conducted to explore the neural time course of the priming process (e.g., Zhang et al., 2006, 2010) or the interaction of valence and arousal on neural correlates of a typical affective priming study (e.g., Zhang et al., 2012). As mentioned before, emotional pictures are also used as primes in affect misattribution paradigms (e.g., Hashimoto et al., 2012). In this study, primes were presented for 75 ms. As all of the mentioned presentation times of emotional pictures lie in the range of 75–200 ms, we decided to capture the lower end of short presentation times within the supra-liminal spectrum by presenting emotional pictures for only 200 ms.

In the group of emotional paradigms using long stimulus presentation times, one class of paradigms, that turned out to be of particular interest to investigating the acquisition and change of affective and emotional responses, are those concerned with the evaluative conditioning effect (EC-effect). The EC-effect refers to changes in the evaluation of a neutral conditioned stimulus (CS) due to its repeated pairing with an affect-laden unconditioned stimulus (US; De Houwer et al., 2001). In these paradigms, the affect-laden US as well as the neutral CS are generally presented supra-liminally with lower mean presentation times in the range of 500–2000 ms. Comparable mean presentation times are also used when investigating affective processing in an attentional blink paradigm (e.g., Smith et al., 2006).

Presenting stimuli for either 200 or 1000 ms thus covers a wide spectrum of presentation times typically used in a variety of paradigms applied in emotion research. We tackle the important question whether the subjective affective responses, elicited in the participants, are comparable across these paradigms. While comparable affective response elicitation across these paradigms would allow for an integration of the resultant findings, modulations of affective response elicitation by duration of exposure would require a more differentiated perspective. In this regard, the current study is an important step in determining the degree to which findings from different experimental paradigms, which are used to study emotional responses, may be integrated.

## Methods

### Participants and design

The study sample consisted of 302 students (190 female; *M*_age_ = 22.55, *SD*_age_ = 2.99) from diverse disciplines (psychology = 57.14%; pedagogy = 15.28%; business studies = 8.31%; others = 19.27%) of the University of Trier. Participants were recruited with the help of posters and flyers including inter alia, information on the compensation, participants' task (i.e., picture evaluation), and some exemplary pictures. Thus, participants were already informed about the real purpose of the study before their actual participation. Moreover, participants signed a consent form before participation. In this regard, the study fully complied with the ethics regulations of our university and was exempt from a formal ethics application.

Students received either 5 Euros or course credit in exchange for their participation. Participants were randomly assigned to the conditions of a 4 (Picture subset: 1 vs. 2 vs. 3 vs. 4) × 2 (Block: valence rating first vs. arousal rating first) × 2 (Duration of presentation: 200 vs. 1000 ms) × 2 (Valence: positive vs. negative) × 2 (Arousal: low vs. high) mixed-factorial design with between-participants variation on the first three factors. As standardized effect size, we computed partial η^2^ (Olejnik and Algina, 2000).

### Stimulus material

A stimulus pool was built with pictures found on the internet with various degrees of valence and arousal. The pre-selection of pictures from the internet was determined by their representativeness for the emotional contents mirroring the four possible combinations of high and low valence, and high and low arousal (i.e., positive/low arousal, positive/high arousal, negative/low arousal, and negative/high arousal). For each of these categories, a team of four student research assistants as well as the authors searched ~120 pictures within the internet matching the respective emotional content. After this pre-selection, 60 pictures from each content set were selected, which seemed to be most representative of the targeted content and were also comparable with regard to their perceptual features (e.g., size of the depicted persons, picture resolution etc.). Accordingly, the final stimulus pool comprised 240 colored and black-and-white pictures, which were classified by their valence (positive or negative) and their arousal (low vs. high) content into four groups of 60 pictures each (for a detailed description as well as statistical values of all pictures see Supplementary Tables S1–S4). Stimuli were selected from the internet in order to guarantee that pictures were largely unknown to the participants, which should counteract knowledge-based distortions of participants' spontaneous evaluations of the pictures. All pictures were adjusted to be of roughly the same size. In particular, height of the pictures was fixed to 300 px (~79.375 mm) and width of pictures to 400 px (~105.833 mm). The entire experiment was programmed and presented in E-Prime 2.0 (Schneider et al., 2002). Stimuli were presented on a 19-inch LCD-screen with a screen refresh rate of 60 Hz and a color depth of 32 Bit.

### Procedure

Upon entering the laboratory, participants were welcomed by the experimenter and led into a soundproof and air-conditioned cubicle with a chair and a PC. Participants were seated in front of the screen at a distance of 60 cm. At the beginning of the procedure participants were told that they were going to be presented with pictures for a short duration of time and that they had to rate these pictures. Because of the number of the stimulus material that had to be rated, the pool was quartered. The separation results in four sets of 60 pictures with each of the aforementioned groups containing 15 pictures each (i.e., positive/low arousal, positive/high arousal, negative/low arousal, and negative/high arousal). Participants were randomly assigned to one of the four sets and rated this respective subset of the stimulus pool. Moreover, half of the participants were randomly assigned to one of two duration conditions. Half of the participants were shown the stimuli for 200 ms whereas the other half were shown the stimuli for 1000 ms^1^. Before each of the two rating tasks started (i.e., one for valence and one for arousal), four learning trials were presented to familiarize the participants with the task. On each trial, the target stimulus was presented at the top center of the screen above a graphic rating scale of 200 mm length. While the target stimuli were only presented for a short amount of time (i.e., 200 or 1000 ms), the graphic rating scale remained on the screen until participants had made their evaluation. Participants could indicate their evaluation by dragging a slider with their mouse cursor to a new position on the scale. In the valence rating task participants were asked if the presented picture felt positive or negative to them. The left end of the graphic scale was labeled “negative” and the right end was labeled “positive.” Beyond that no further division of the scale was used. The computer program recorded negative judgments on the left side from −1 to −100, and positive judgments on the right side from +1 to +100. The neutral midpoint of the scale (0) served as the starting position for each judgment. In the arousal rating task, participants had to rate if the presented pictures were creating a sensation of calmness (low arousal) or a sensation of activation (high arousal). Endpoints of the scale were labeled “calmness” on the left, and activation on the right. In order to ensure that participants would focus on their physical reactions when making those assessments and thus their actual arousal response, we provided as a further anchor on this decision a Self-Assessment Manikin (SAM; Bradley and Lang, 1994) scale for arousal. This scale was presented above the graphic rating scale in each trial. Comparable to the valence rating task the computer program recorded calmness judgments on the left side from −1 to −100, and activation judgments on the right side from +1 to +100. After the rating of a stimulus, participants had to click on a “next” button to proceed to the next trial. The order of stimulus presentation was randomized for each participant. Participants rated each stimulus of their assigned subset on both dimensions. The order of valence and arousal rating was counterbalanced between participants. After completing all trials, participants were thanked, debriefed, and then awarded their compensation.

## Results

Mean scores of rated valence were composed by averaging over valence ratings of each stimulus in each group of valence and arousal dimension, and over sets within these groups. Mean scores of rated arousal were composed the same way over arousal ratings. Those average scores were analyzed by a 2 (Duration: 200 vs. 1000 ms) × 2 (Valence: positive vs. negative) × 2 (Arousal: low vs. high) mixed models analysis of variance with between variation on the first and within variation on the second and third factor^2^. All sets had almost equal numbers of participants (*N*_Set1_ = 77, *N*_Set2_ = 76, *N*_Set3_ = 75, *N*_Set4_ = 74) that rated the pictures within the set.

### Valence rating

**Figure 1:**
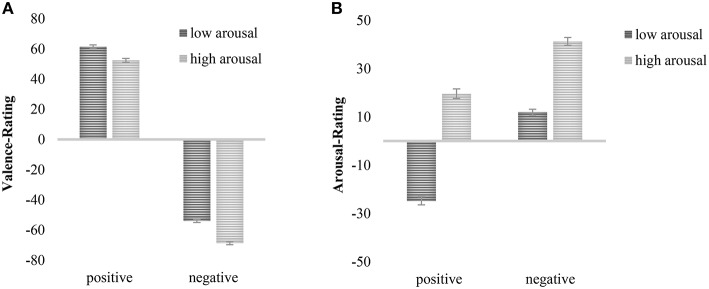
**(A)** Interaction of valence and arousal on valence ratings. **(B)** Interaction of valence and arousal on arousal ratings. Mean values plus standard error reported.

The analysis for valence ratings revealed the expected significant main effect for valence, *F*_(1, 300)_ = 3968.22, *p* < 0.001, η_*p*_^2^ = 0.93, indicating that stimuli categorized as positive were rated more positive than stimuli of negative categorization (*M*_pos_ = 56.91, *SD*_pos_ = 19.48, *M*_neg_ = −61.4, *SD*_neg_ = 16.14). This finding can be seen as a manipulation check indicating that prior categorization of valence is empirically supported. An interaction between valence and duration reached marginal significance, *F*_(1, 300)_ = 3.85, *p* < 0.10, η_*p*_^2^ = 0.01 (negative: *M*_200ms_ = −58.14, *SD*_200ms_ = 18.09, *M*_1000ms_ = −64.64, *SD*_1000ms_ = 16.37; positive: *M*_200ms_ = 56.48, *SD*_200ms_ = 21.93, *M*_1000ms_ = 57.35, *SD*_1000ms_ = 21.95). *Post-hoc* analyses revealed that negative pictures were evaluated more negatively in the 1000 ms condition as compared to the 200 ms condition, *t*_(300)_ = 3.47, *p* < 0.001, *d* = 0.40. However, there were no differences in evaluation for positive images, *t*_(300)_ = −0.39, *p* = 0.698, *d* = 0.04. Furthermore, a significant main effect of arousal was observed, *F*_(1, 300)_ = 333.12, *p* < 0.001, η_*p*_^2^ = 0.53, showing that stimuli of low arousal were rated more positive than stimuli of high arousal (*M*_low_ = 3.72, *SD*_low_ = 8.46, *M*_high_ = −8.2, *SD*_high_ = 10.04). The main effect was qualified by an interaction with duration, *F*_(1, 300)_ = 6.58, *p* < 0.05, η_*p*_^2^ = 0.02, showing that high arousing pictures were rated more unpleasant when they were presented for 1000 ms than for 200 ms, *t*_(300)_ = 3.89, *p* < 0.001, *d* = 0.45 (*M*_1000ms_ = −10.44, *SD*_1000ms_ = 19.25 and *M*_200ms_ = −5.95, *SD*_200ms_ = 19.98, respectively), whereas the low arousal condition did not show a differentiation, *t*_(300)_ = 1.16, *p* = 0.25, *d* = 0.14. There was also a significant main effect of duration, *F*_(1, 300)_ = 11.09, *p* < 0.001, η_*p*_^2^ = 0.04, with stimuli presented for 200 ms being rated more positive than stimuli presented for 1000 ms (*M*_200ms_ = −0.83, *SD*_200ms_ = 7.35, *M*_1000ms_ = −3.65, *SD*_1000ms_ = 7.45). Moreover, there was a significant interaction between valence and arousal, *F*_(1, 300)_ = 16.54, *p* < 0.001, η_*p*_^2^ = 0.05 (Figure 1A) indicating that negative pictures of high arousal were evaluated more negatively than negative low arousing ones (*M*_high_ = −68.74, *SD*_high_ = 17.25 and *M*_low_ = −53.96, *SD*_low_ = 17.86, respectively), *t*_(301)_ = −20.84, *p* < 0.001, *d* = 2.4, and positive low arousing pictures were rated more pleasant than positive high arousing pictures (*M*_low_ = 61.42, *SD*_low_ = 21.44 and *M*_high_ = 52.40, *SD*_high_ = 22.43, respectively), *t*_(301)_ = 7.73, *p* < 0.001, *d* = 0.89.

### Arousal rating

The analysis for arousal ratings revealed a significant main effect of arousal indicating that stimuli categorized to contain high arousal were rated more arousing than stimuli categorized to be of low arousal, *F*_(1, 300)_ = 1007.34, *p* < 0.001, η_*p*_^2^ = 0.77, (*M*_high_ = 30.43, *SD*_high_ = 18.84 and *M*_low_ = −6.44, *SD*_low_ = 18.49, respectively). There was also a significant main effect of valence, *F*_(1, 300)_ = 265.66, *p* < 0.001, η_*p*_^2^ = .47, (*M*_pos_ = −2.61, *SD*_pos_ = 24.38, *M*_neg_ = 26.6, *SD*_neg_ = 19.58), indicating that negative pictures were evaluated as more arousing than positive pictures. Moreover, there was an interaction of valence and duration, *F*_(1, 300)_ = 4.04, *p* < 0.05, η_*p*_^2^ = 0.01, (negative: *M*_200ms_ = 24.56, *SD*_200ms_ = 1.58, *M*_1000ms_ = 28.65, *SD*_1000ms_ = 1.6; positive: *M*_200ms_ = −1.05, *SD*_200ms_ = 1.97, *M*_1000ms_ = −4.17, *SD*_1000ms_ = 2.00). *Post-hoc* analyses revealed marginally significant higher arousal ratings for negative stimuli that were presented for 1000 ms than 200 ms, *t*_(300)_ = 1.81, *p* = 0.076, *d* = 0.21, and no effect of duration for positive stimuli, *t*_(300)_ = 1.12, *p* = 0.268, *d* = 0.13. Finally, there was a significant interaction of valence and arousal, *F*_(1, 300)_ = 26.46, *p* < 0.001, η_*p*_^2^ = 0.15 (Figure 1B). *Post hoc* analyses revealed that for both low and high arousing pictures, pictures of positive valence were evaluated less arousing than those of negative valence, *t*_(301)_ = −16.60, *p* < 0.001, *d* = 1.38 and *t*_(301)_ = −11.77, *p* < 0.001, *d* = 0.88, respectively (positive: *M*_low_ = −24.77, *SD*_low_ = 30.61, *M*_high_ = 19.60, *SD*_high_ = 27.69; negative: *M*_low_ = 11.90, *SD*_low_ = 21.94, *M*_high_ = 41.25, *SD*_high_ = 21.28).

### Reliability analysis

**Figure 2:**
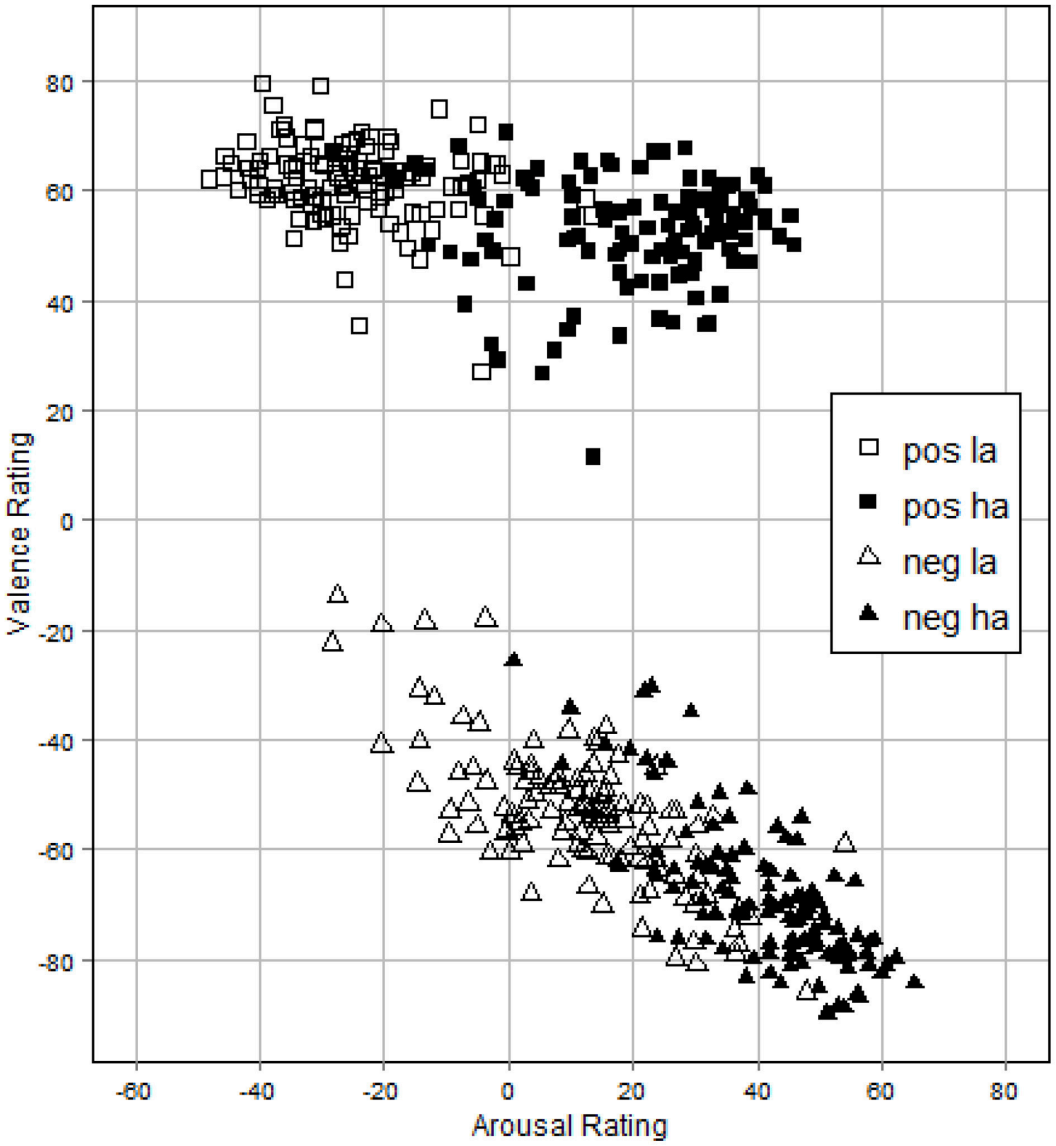
**Correlation plot of valence and arousal ratings for each picture**. Values indicate mean ratings for every stimulus. pos la, positive/low arousal; pos ha, positive/high arousal; neg la, negative/low arousal; neg ha, negative/high arousal.

To check for the internal consistency of items within the different sets and thus their comparability we calculated the intraclass correlation coefficient (ICC) by making use of a two-way random model. Indices were computed for each group (positive/low arousing, positive/high arousing, negative/low arousing, and negative/high arousing) of 15 pictures within each of the four sets and for both duration conditions. The analysis was done for valence as well as arousal ratings. As shown in Tables 1, 2, the results indicate a good internal consistency for all groups within sets with ICC ranging from 0.81 to 0.96. For the duration conditions ICC ranged from 0.76 to 0.97. Figure 2 shows a correlation plot of valence and arousal ratings.

**Table 1:** **Intraclass correlation coefficients (ICC) of the valence rating procedure**.

		**pos la**		**pos ha**		**neg la**		**neg ha**	
Set 1	200 ms	0.96	0.95	0.95	0.94	0.90	0.89	0.90	0.88
	1000 ms		0.96		0.96		0.91		0.88
Set 2	200 ms	0.92	0.94	0.88	0.88	0.85	0.84	0.85	0.87
	1000 ms		0.88		0.88		.85		0.77
Set 3	200 ms	0.94	0.90	0.94	0.94	0.91	0.91	0.84	0.78
	1000 ms		0.97		0.94		0.91		0.92
Set 4	200 ms	0.93	0.95	0.96	0.96	0.86	0.84	0.88	0.89
	1000 ms		0.90		0.95		0.87		0.87

*pos la, positive/low arousal; pos ha, positive/high arousal; neg la, negative/low arousal; neg ha, negative/high arousal*.

**Table 2:** **Intraclass correlation coefficients (ICC) of the arousal rating procedure**.

		**pos la**		**pos ha**		**neg la**		**neg ha**	
Set 1	200 ms	0.94	0.93	0.93	0.94	0.91	0.93	0.92	0.92
	1000 ms		0.94		0.91		0.89		0.92
Set 2	200 ms	0.87	0.90	0.88	0.89	0.83	0.83	0.81	0.80
	1000 ms		0.88		0.86		0.84		0.83
Set 3	200 ms	0.94	0.94	0.92	0.93	0.86	0.87	0.86	0.78
	1000 ms		0.94		0.91		0.85		0.91
Set 4	200 ms	0.91	0.92	0.95	0.96	0.87	0.85	0.83	0.76
	1000 ms		0.90		0.94		0.88		0.85

*pos la, positive/low arousal; pos ha, positive/high arousal; neg la, negative/low arousal; neg ha, negative/high arousal*.

## Discussion

Our main objective was to analyze the temporal robustness of affective responses. Specifically, the study tested the temporal robustness of affective responses in a range of presentation times typically used across many paradigms in emotion research.

The valence ratings as well as arousal ratings revealed the expected main effects of valence and arousal. This finding validates the pre-categorization of the pictures on the affective response dimensions, valence, and arousal. On both rating dimensions, main effects of the opposite dimension (e.g., valence on arousal ratings, and vice versa) were significant. The two significant main effects formed an interaction on both valence and arousal ratings. In addition, all sets were tested for their internal consistency for both the 200 and the 1000 ms condition. ICCs ranged between 0.76 and 0.97 indicating a good to very good consistency of sets and groups for both presentation time conditions.

Most important for the purpose of the current study, however, were the results regarding the *duration* factor. In particular, the main result was that affective responses were quite robust irrespective of presentation time. A closer look, however, showed that this pattern was somewhat more complex, as indicated by the interaction of duration and arousal, and duration and valence on valence ratings. With regard to the first interaction effect it turned out that high arousing pictures elicit more negative ratings if they are presented for longer time intervals while the presentation time did not influence the valence ratings of low arousing pictures. Complementing the finding that high arousal results in more negative evaluations when presented for 1000 ms as compared to 200 ms, the interaction between valence and duration showed that negative images, but not positive images, were evaluated slightly more negative in the 1000 ms condition. Moreover, there turned out to be a comparable interaction between valence and duration for arousal ratings; that is, negative stimuli elicited more arousing ratings when they were presented for 1000 ms as compared to 200 ms. In sum, these results indicate that the impact of high arousing stimuli on cognition, which are naturally negative in valence, increases with exposure duration. To be more precise, the more the exposure duration to negative high arousing events increases, the more the evaluation of those events and the activation of the alerting system will increase. Thus, these results reflect the high sensitivity of our cognitive system in processing emotional events with high affordances regarding action control. That the involvement of the alerting system might depend on exposure duration has also been proposed in previous research concerned with the temporal constraints of the motivational processing of emotional stimuli (Codispoti et al., 2001). However, insofar as the authors did not experimentally vary presentation duration, this notion needed further experimental evidence which our findings might provide. However, it has to be noted that these interaction effects with duration explained only little variance (η_*p*_^2^ was 0.01 or 0.02). Thus, they complement but do not disqualify our main finding that affective response elicitation is relatively stable across different presentation times. In turn, this relatively high temporal robustness indicates that the use of emotional pictures in paradigms comprising stimulus presentation times between 200 and 1000 ms should evoke comparable emotional responses. These findings have important implications for social cognitive research that investigates emotional and affective processing. When investigating emotional and affective processing within the supra-liminal spectrum, presentation times of the affective material strongly differ between paradigms. Therefore, it might be argued that, for instance, affective responses in an affective priming task are not comparable to those elicited in an evaluative conditioning paradigm. That is, the comparability of affective responses elicited by the same material might be questioned. The results of the current study counteract such an argumentation by providing relatively strong evidence to the assumption that affective response elicitation is robust across presentation times ranging between 200 and 1000 ms. Moreover, these findings are also relevant to many cognitive psychology studies because they are also greatly dependent on temporally robust affective picture material. Whether these studies use an altered task switch paradigm (Braem et al., 2013), dot probe tasks (Kappenman et al., 2015), stroop-like paradigms (Padmala et al., 2011), a visual discrimination task (Shafer et al., 2012), or a varied flanker task, all these approaches use shortly presented emotional pictures (200–500 ms).

Nonetheless, it should be noted that our results are restricted to affective responses at the lower end of the supra-liminal spectrum. In order to further our understanding on affective response elicitation across the whole temporal spectrum, future research should also include shorter presentation times like 20–40 ms. Specifically, including shorter presentation times might broaden the scope of our findings to masked or subliminal procedures. Likewise, future research should address the question whether the interaction pattern found in the current study becomes more prominent when further increasing the duration of exposure. This would be especially important with regard to the nature of the motivational and attentional processes involved in affective response elicitation. In particular, one might determine inasmuch the operation of these processes actually follows an all-or-none principle or a more context sensitive principle.

Moreover, one might question whether our findings do also apply to pictures from the more established databases (e.g., IAPS, EmoPics, GAPED), because we only tested self-selected pictures from the internet. Even though pictures from at least some of the established picture databases have also been selected from the internet (e.g., GAPED database and also the EmoPics database), the reliability of our findings regarding other picture databases needs to be tested empirically. Additionally, the generalizability of our findings to the larger population should be investigated in future research by making use of a more heterogeneous sample (i.e., stronger variability with regard to educational background, age, etc.). In this context replicating our study with picture material from more established databases as well as with a more representative sample should be worthwhile goals for future research.

In conclusion, since it is an operational need of emotional research to ensure a stable affective response elicitation across paradigms, the importance of an empirical test on the temporal robustness of affective responses cannot be overstated.

## Author contributions

The initial idea for the methodological hypothesis was conceived by CP and KB and was further refined with the help of CF and EW. Data aggregation and analysis was conducted by CP and KB. All authors contributed to the interpretation of the data and writing the manuscript. All authors approved the final version of the manuscript for submission and take responsibility for its content.

## Funding

The German Science Foundation supported this research through grants WA 1344/9-1 and FR 2133/10-1 to EW and CF.

### Conflict of interest statement

The authors declare that the research was conducted in the absence of any commercial or financial relationships that could be construed as a potential conflict of interest.

## Footnotes

^1^Presentation duration was varied between subjects in order to keep inter-rater variability as low as possible while at the same time ensuring a constantly high level of motivated attention to the task. Specifically, manipulating presentation duration within-subjects would either have required a doubling of the absolute number of ratings for each participant or a further splitting of the stimulus pool. In any case inter-rater variability or motivated attention to the task would vary thereby endangering the interpretability of our results.

^2^Due to a technical problem, one picture of positive valence and low arousal out of the third set was not presented in valence rating and therefore had to be excluded from both valence and arousal ratings. To ascertain this decision, both analyses (picture included vs. excluded) were computed showing that results of both analyses did not differ significantly.

## References

[B1] Bradley M. M. (2009). Natural selective attention: orienting and emotion. Psychophysiology 46, 1–11. 10.1111/j.1469-8986.2008.00702.x 18778317 PMC3645482

[B2] Bradley M. M. Hamby S. Löw A. Lang P. J. (2007). Brain potentials in perception: picture complexity and emotional arousal. Psychophysiology 44, 364–373. 10.1111/j.1469-8986.2007.00520.x 17433095

[B3] Bradley M. M. Lang P. J. (1994). Measuring emotion: the self-assessment manikin and the semantic differential. J. Behav. Ther. Exp. Psychiatry 25, 49–59. 10.1016/0005-7916(94)90063-9 7962581

[B4] Braem S. King J. A. Korb F. M. Krebs R. M. Notebaert W. Egner T. (2013). Affective modulation of cognitive control is determined by performance-contingency and mediated by ventromedial prefrontal and cingulate cortex. J. Neurosci. 33, 16961–16970. 10.1523/JNEUROSCI.1208-13.2013 24155301 PMC3807025

[B5] Carroll J. B. Osgood C. E. Suci G. J. Tannenbaum P. H. (1959). The measurement of meaning. Language 35, 197–237. 10.2307/41133512729139

[B6] Codispoti M. Bradley M. M. Lang P. J. (2001). Affective reactions to briefly presented pictures. Psychophysiology 38, 474–478. 10.1111/1469-8986.3830474 11352135

[B7] Dan-Glauser E. S. Scherer K. R. (2011). The Geneva affective picture database (GAPED): a new 730-picture database focusing on valence and normative significance. Behav. Res. Methods 43, 468–477. 10.3758/s13428-011-0064-1 21431997

[B8] De Houwer J. Thomas S. Baeyens F. (2001). Associative learning of likes and dislikes: a review of 25 years of research on human evaluative conditioning. Psychol. Bull. 127, 853–869. 10.1037/0033-2909.127.6.853 11726074

[B9] Drace S. Efendic E. Kusturica M. Landzo L. (2013). Cross-cultural validation of the “International affective picture system” (IAPS) on a sample from Bosnia and Herzegovina. Psihologija 46, 17–26. 10.2298/PSI1301017D

[B10] Dufey M. Fernández A. M. Mayol R. (2011). Adding Support to Cross-Cultural Emotional Assessment: Validation of the International Affective Picture System in a Chilean Sample. Available online at: http://www.redalyc.org/articulo.oa?id=64722451016 (Accessed May 24, 2015).

[B11] Eder A. B. Leuthold H. Rothermund K. Schweinberger S. R. (2012). Automatic response activation in sequential affective priming: an ERP study. Soc. Cogn. Affect. Neurosci. 7, 436–445. 10.1093/scan/nsr033 21642351 PMC3324576

[B12] Hamm A. O. Schupp H. T. Weike A. I. (2003). Motivational organization of emotions: autonomic changes, cortical responses, and reflex modulation, in Handbook of Affective Sciences, eds Davidson R. J. Scherer K. Goldsmith H. H. (Oxford, UK: Oxford University Press), 187–211.

[B13] Hashimoto Y. Minami T. Nakauchi S. Koenig T. (2012). Electrophysiological differences in the processing of affect misattribution. PLoS ONE 7:e49132. 10.1371/journal.pone.0049132 23145097 PMC3492313

[B14] Hermans D. De Houwer J. Eelen P. (1994). The affective priming effect: automatic activation of evaluative information in memory. Cogn. Emot. 8, 515–533. 10.1080/02699939408408957

[B15] Huang Y.-M. Baddeley A. Young A. W. (2008). Attentional capture by emotional stimuli is modulated by semantic processing. J. Exp. Psychol. 34, 328–339. 10.1037/0096-1523.34.2.328 18377174

[B16] Kappenman E. S. MacNamara A. Proudfit G. H. (2015). Electrocortical evidence for rapid allocation of attention to threat in the dot-probe task. Soc. Cogn. Affect. Neurosci. 10, 577–583. 10.1093/scan/nsu098 25062842 PMC4381248

[B17] Kuppens P. Tuerlinckx F. Russell J. A. Barrett L. F. (2013). The relation between valence and arousal in subjective experience. Psychol. Bull. 139, 917–940. 10.1037/a0030811 23231533

[B18] Lang P. Bradley M. M. Cuthbert B. (2008). International Affective Picture System (IAPS): Affective Ratings of Pictures and Instruction Manual. Technical Report A-8, Gainesville, FL: University of Florida.

[B19] Lang P. J. Bradley M. M. Cuthbert B. N. (1997). Motivated attention: affect, activation, and action, in Attention and Orienting, eds Lang P. J. Simons R. F. Balaban M. (Mahwah, NJ: Erlbaum), 97–135.

[B20] Lang P. J. Greenwald M. K. Bradley M. M. Hamm A. O. (1993). Looking at pictures: affective, facial, visceral, and behavioral reactions. Psychophysiology 30, 261–273. 10.1111/j.1469-8986.1993.tb03352.x 8497555

[B21] Marchewka A. Żurawski Ł. Jednoróg K. Grabowska A. (2014). The Nencki Affective Picture System (NAPS): introduction to a novel, standardized, wide-range, high-quality, realistic picture database. Behav. Res. Methods 46, 596–610. 10.3758/s13428-013-0379-1 23996831 PMC4030128

[B22] Olejnik S. Algina J. (2000). Measures of effect size for comparative studies: applications, interpretations, and limitations. Contemp. Educ. Psychol. 25, 241–286. 10.1006/ceps.2000.1040 10873373

[B23] Osgood C. E. (1952). The nature and measurement of meaning. Psychol. Bull. 49, 197–237. 10.1037/h0055737 14930159

[B24] Padmala S. Bauer A. Pessoa L. (2011). Negative emotion impairs conflict-driven executive control. Front. Psychol. 2:192. 10.3389/fpsyg.2011.00192 21886635 PMC3154405

[B25] Russell J. A. (2003). Core affect and the psychological construction of emotion. Psychol. Rev. 110, 145–172. 10.1037/0033-295X.110.1.145 12529060

[B26] Schneider W. Eschman A. Zuccolotto A. (2002). E-Prime User's Guide. Pittsburgh, PA: Psychology Software Tools Inc.

[B27] Shafer A. T. Matveychuk D. Penney T. O'Hare A. J. Stokes J. Dolcos F. (2012). Processing of emotional distraction is both automatic and modulated by attention: evidence from an event-related fMRI investigation. J. Cogn. Neurosci. 24, 1233–1252. 10.1162/jocn_a_00206 22332805 PMC4491634

[B28] Smith S. D. Most S. B. Newsome L. A. Zald D. H. (2006). An emotion-induced attentional blink elicited by aversively conditioned stimuli. Emotion 6, 523–527. 10.1037/1528-3542.6.3.523 16938093

[B29] Spruyt A. Hermans D. De Houwer J. Eelen P. (2002). On the nature of the affective priming effect: affective priming of naming responses. Soc. Cogn. 20, 227–256. 10.1521/soco.20.3.227.21106

[B30] Spruyt A. Hermans D. De Houwer J. Vandromme H. Eelen P. (2007). On the nature of the affective priming effect: effects of stimulus onset asynchrony and congruency proportion in naming and evaluative categorization. Mem. Cognit. 35, 95–106. 10.3758/BF03195946 17533884

[B31] Wentura D. Degner J. (2010). A practical guide to sequential priming and related tasks, in Handbook of Implicit Social Cognition. Measurement, Theory, and Applications, eds Gawronski B. Payne B. K. (New York, NY: Guilford Press), 95–116.

[B32] Wentura D. Rothermund K. (2014). Priming is not Priming is not Priming. Soc. Cogn. 32, 47–67. 10.1521/soco.2014.32.supp.47

[B33] Wessa M. Kanske P. Neumeister P. Bode K. Heissler J. Schönfelder S. (2010). EmoPics: subjektive und psychophysiologische Evaluationen neuen Bildmaterials für die klinisch-bio-psychologische Forschung. Z. Klin. Psychol. Psychother. 39(Suppl. 1/11), 77.

[B34] Wundt W. (1896). Grundriß der Psychologie. Leipzig: Engelmann.

[B35] Zhang Q. Kong L. Jiang Y. (2012). The interaction of arousal and valence in affective priming: behavioral and electrophysiological evidence. Brain Res. 1474, 60–72. 10.1016/j.brainres.2012.07.023 22820299 PMC3694405

[B36] Zhang Q. Lawson A. Guo C. Jiang Y. (2006). Electrophysiological correlates of visual affective priming. Brain Res. Bull. 71, 316–323. 10.1016/j.brainresbull.2006.09.023 17113962 PMC1783676

[B37] Zhang Q. Li X. Gold B. T. Jiang Y. (2010). Neural correlates of cross-domain affective priming. Brain Res. 1329, 142–151. 10.1016/j.brainres.2010.03.021 20298681 PMC2857548

