# Supplementary material for: Timeless: A Large Sample Study on the Temporal Robustness of Affective Responses
Source: Front Psychol. 2016 Jun 1;7:841. doi: 10.3389/fpsyg.2016.00841 (PMC4887497; doi:10.3389/fpsyg.2016.00841)
Supplement: Supplementary file 1 [file Table1.pdf]

Supplementary Material

Table S1

Description and statistical values of the positive low arousing (pl) pictures.

Description	Picnum	Valence-Rating				Arousal-Rating			
		M (SD)	skew	kurt	N	M (SD)	skew	kurt	N
baby smile	pl_001	55,77 (37,24)	-1,189	2,976	77	-24,78 (42,90)	0,334	-0,323	77
babies smile	pl_002	67,79 (28,35)	-0,411	-1,046	77	-35,06 (42,99)	0,131	-1,121	77
baby smile	pl_003	67,87 (26,77)	-0,315	-0,88	77	-30,51 (46,99)	0,303	-0,889	77
family happy	pl_004	65,45 (28,37)	-0,359	-0,798	77	-36,22 (45,75)	0,54	-0,371	77
children smile	pl_005	64,97 (28,51)	-0,289	-1,058	77	-20,29 (46,60)	0,205	-0,73	77
family beach	pl_006	62,51 (30,36)	-0,323	-1,045	77	-11,31 (49,16)	0,017	-0,927	77
children beach	pl_007	60,61 (28,65)	-0,273	-0,727	77	-42,66 (39,12)	0,574	-0,3	77
friends happy	pl_008	58,35 (33,22)	-1,016	2,634	77	-36,66 (38,64)	0,428	0,064	77
friends happy[bw]	pl_009	58,45 (28,55)	0,028	-0,994	77	-37,61 (39,19)	0,5	-0,253	77
child smile	pl_010	60,29 (28,37)	-0,117	-0,85	77	-30,36 (39,99)	0,446	-0,303	77
children happy	pl_011	71,58 (26,92)	-0,757	-0,27	77	-37,17 (38,65)	0,228	-0,995	77
friends happy	pl_012	52,64 (33,12)	-0,419	-0,135	77	-15,26 (43,31)	0,109	-0,583	77
child smile	pl_013	70,96 (28,39)	-1,246	2,151	77	-22,90 (46,84)	0,123	-1,209	77
children smile	pl_014	65,19 (26,70)	-0,281	-0,962	77	-34,78 (41,15)	0,477	-0,498	77
child happy	pl_015	60,38 (31,37)	-0,276	-1,187	77	-20,69 (46,93)	0,18	-0,949	77
children smile	pl_016	57,43 (28,29)	-0,085	-0,731	76	-20,89 (42,35)	0,287	-0,607	76
children happy	pl_017	65,32 (31,15)	-1,256	2,46	76	-12,95 (42,99)	0,078	-0,805	76
adults happy	pl_018	41,08 (29,15)	-0,028	-0,342	76	-16,93 (37,19)	0,204	-0,605	76
family happy	pl_019	67,12 (29,22)	-0,864	0,631	76	-20,82 (40,59)	0,253	-0,387	76
family happy	pl_020	64,89 (28,03)	-0,52	-0,049	76	-21,79 (44,47)	0,384	-0,389	76
friends happy	pl_021	63,88 (26,75)	-0,459	-0,634	76	-1,80 (41,64)	-0,448	-0,361	76
friends happy	pl_022	63,32 (29,06)	-0,436	-0,393	76	-3,42 (42,71)	0,142	-0,003	76
friends happy	pl_023	55,14 (26,56)	0,125	-1,058	76	-13,71 (36,43)	0,193	-0,281	76
friends happy	pl_024	51,75 (31,44)	-0,682	1,889	76	-1,87 (41,77)	0,165	0,004	76

children happy	pl_025	65,26 (29,20)	-0,884	0,849	76	-28,83 (39,64)	0,542	0,233	76
mom baby smile	pl_026	67,75 (29,66)	-0,871	0,355	76	-23,18 (47,21)	0,306	-0,511	76
mom child smile	pl_027	59,89 (29,44)	-0,639	0,998	76	-35,84 (35,99)	0,729	0,547	76
puppy	pl_028	57,92 (32,49)	-0,587	-0,168	76	-31,57 (52,14)	0,775	-0,347	76
hamster	pl_029	52,93 (34,37)	-1,003	2,979	76	-27,80 (48,94)	0,799	0,098	76
child happy	pl_030	56,76 (28,93)	-0,181	-0,62	76	-20,47 (37,91)	0,304	-0,073	76
child smile	pl_031	61,91 (27,09)	-0,296	-0,712	75	-38,35 (39,15)	0,484	-0,383	75
children smile	pl_032	65,99 (30,00)	-0,802	0,301	75	-35,09 (43,55)	0,703	-0,026	75
children happy	pl_033	47,04 (39,64)	-1,444	3,617	75	-27,55 (42,00)	0,339	-0,499	75
children happy	pl_034	69,96 (27,62)	-0,558	-1,07	75	-36,67 (41,95)	0,46	-0,771	75
children smile	pl_035	60,57 (34,46)	-1,355	3,24	75	-33,49 (43,42)	0,638	0,046	75
child smile	pl_036	61,69 (27,15)	-0,473	-0,435	75	-33,44 (42,52)	0,347	-0,556	75
babies happy	pl_037	69,60 (28,31)	-1,081	1,166	75	-27,57 (52,96)	0,52	-0,597	75
mom baby happy	pl_038	66,57 (26,46)	-0,772	0,883	75	-31,16 (51,88)	0,636	-0,511	75
mom baby happy	pl_039	62,60 (27,88)	-0,581	0,471	75	-34,48 (44,17)	0,389	-0,18	75
child smile	pl_040								
family happy	pl_041	68,73 (30,90)	-1,326	2,558	75	-41,15 (45,91)	1,029	0,915	75
child smile	pl_042	54,56 (32,30)	-0,574	0,06	75	-33,16 (40,14)	0,241	-0,595	75
friends happy	pl_043	60,37 (29,47)	-0,823	0,698	75	-25,64 (42,75)	-0,011	-1,012	75
friends happy	pl_044	68,21 (25,96)	-0,602	-0,366	75	-15,99 (50,19)	0,125	-0,905	75
mom child happy	pl_045	62,19 (27,00)	-0,399	-0,798	75	-42,67 (40,60)	0,463	-0,542	75

Description	Picnum	Valence-Rating				Arousal-Rating			
		M (SD)	skew	kurt	N	M (SD)	skew	kurt	N
children happy	pl_046	63,15 (28,70)	-0,816	0,743	74	-5,57 (47,70)	-0,009	-0,833	74
couple happy	pl_047	62,58 (26,74)	-0,36	-0,559	74	-24,31 (38,80)	0,313	-0,523	74
couple happy	pl_048	62,23 (29,85)	-0,656	0,274	74	-17,41 (39,93)	0,232	-1,042	74
couple happy	pl_049	60,95 (25,24)	-0,363	-0,326	74	-7,09 (49,07)	-0,018	-0,971	74
couple happy	pl_050	63,28 (25,25)	0,013	-1,231	74	-22,27 (43,32)	0,511	-0,432	74
children happy	pl_051	54,53 (30,44)	-0,521	0,639	74	-19,12 (41,54)	0,277	-0,743	74
child smile	pl_052	63,81 (27,57)	-0,483	-0,626	74	-28,96 (43,98)	0,549	0,01	74
baby happy	pl_053	63,97 (31,37)	-1,034	1,254	74	-10,89 (50,41)	-0,02	-0,918	74

friends smile	pl_054	56,23 (26,89)	-0,156	-0,459	74	-29,11 (42,28)	0,684	0,276	74
children happy	pl_055	53,80 (31,83)	-0,891	2,455	74	-18,73 (45,16)	0,375	-0,713	74
mom baby happy	pl_056	65,62 (32,97)	-1,25	1,614	74	-30,42 (45,47)	0,455	-0,589	74
friends excited	pl_057	56,96 (26,78)	-0,126	-0,717	74	12,95 (43,48)	-0,49	-0,101	74
children smile	pl_058	58,61 (25,27)	-0,216	-0,219	74	-30,28 (44,49)	0,648	0,116	74
couple happy	pl_059	61,80 (25,98)	-0,079	-1,085	74	-22,27 (43,40)	0,227	-1,191	74
couple happy	pl_060	61,12 (26,69)	-0,212	-0,574	74	-24,07 (42,88)	0,353	-0,497	74

Note. M = Mean, SD = Standard Deviation, skew = skewness, kurt = kurtosis, N = number of participants rating each picture, pl = positive/low arousal. Picture sets were defined as follows: Set1 = Picnum 001-015; Set2 = Picnum 016-030; Set3 = Picnum 031-045; Set 4 = Picnum 046-060. Due to a technical problem “pl_040” was not presented and rated and was therefore excluded from the stimulus pool.

Table S2

Description and statistical values of the positive high arousing (ph) pictures.

Description	Picnum	Valence-Rating				Arousal-Rating			
		M (SD)	skew	kurt	N	M (SD)	skew	kurt	N
couple naked	ph_001	59,01 (30,69)	-0,142	-1,209	77	32,64 (37,16)	-0,651	0,234	77
couple kiss	ph_002	56,34 (30,26)	0,042	-1,065	77	17,39 (42,53)	-0,459	-0,083	77
female couple[bw]	ph_003	47,91 (37,91)	-0,922	2,252	77	24,62 (40,81)	-0,383	-0,167	77
couple naked	ph_004	63,23 (28,58)	-0,116	-1,214	77	8,99 (43,88)	-0,573	-0,134	77
kiss	ph_005	48,58 (31,99)	-0,052	-0,72	77	-9,49 (38,18)	-0,409	-0,599	77
friends excited	ph_006	64,34 (29,63)	-0,564	-0,624	77	-16,88 (46,86)	0,248	-0,684	77
friends excited	ph_007	61,88 (29,83)	-0,345	-0,949	77	-9,31 (50,16)	0,238	-0,712	77
couple kiss[bw]	ph_008	54,64 (31,91)	-0,183	-0,808	77	20,86 (40,21)	-0,597	0,405	77
couple erotic[bw]	ph_009	56,55 (30,32)	0,145	-1,299	77	35,82 (39,67)	-0,541	0,055	77
friends excited	ph_010	64,44 (28,06)	-0,557	-0,391	77	-23,12 (47,65)	0,333	-0,541	77
couple kiss[bw]	ph_011	54,30 (29,87)	-0,087	-0,949	77	-2,10 (41,11)	-0,038	-0,403	77
friends dancing	ph_012	46,51 (33,67)	-0,01	-0,824	77	17,66 (42,74)	-0,536	0,228	77
couple erotic[bw]	ph_013	54,30 (32,07)	-0,003	-1,056	77	30,52 (40,35)	-0,671	0,845	77
couple erotic[bw]	ph_014	47,87 (34,55)	-0,032	-0,805	77	35,44 (41,00)	-0,77	0,434	77
couple erotic[bw]	ph_015	57,03 (31,08)	0,032	-1,37	77	29,81 (40,66)	-0,674	-0,025	77
friends excited	ph_016	62,70 (29,63)	-0,983	1,365	76	15,58 (40,75)	-0,587	0,046	76
adult excited	ph_017	18,96 (37,86)	-0,518	0,673	76	9,59 (36,83)	-0,44	0,943	76
couple kiss[bw]	ph_018	57,76 (30,53)	-0,541	-0,67	76	34,04 (35,75)	-0,635	0,654	76
couple erotic[bw]	ph_019	35,62 (32,53)	0,043	-0,736	76	29,12 (36,55)	-0,605	0,033	76
concert excited	ph_020	51,91 (32,76)	-0,386	-0,292	76	24,64 (45,83)	-0,621	-0,093	76
female couple[bw]	ph_021	45,68 (33,00)	0,036	-0,832	76	29,79 (37,11)	-0,629	0,432	76
concert excited	ph_022	56,64 (32,39)	-0,696	0,657	76	39,83 (37,93)	-0,956	1,29	76
concert excited	ph_023	50,54 (35,66)	-1,295	3,277	76	38,75 (35,39)	-0,322	-0,074	76
friends excited	ph_024	59,37 (30,65)	-0,928	2,568	76	-,37 (45,58)	-0,116	-0,75	76

couple erotic[bw]	ph_025	48,46 (35,24)	-0,463	-0,053	76	42,41 (30,64)	-0,124	-0,462	76
couple kiss	ph_026	56,45 (26,61)	-0,033	-0,788	76	23,79 (41,22)	-0,547	0,461	76
lingerie model	ph_027	31,70 (31,49)	0,51	-0,313	76	3,89 (43,22)	-0,479	0,245	76
friends excited	ph_028	65,12 (30,60)	-1,121	2,051	76	-2,71 (45,21)	0,077	-0,667	76
female erotic[bw]	ph_029	38,01 (38,60)	-0,277	-0,341	76	31,28 (38,94)	-0,593	0,366	76
couple erotic	ph_030	53,96 (32,16)	-0,125	-1,079	76	37,14 (36,18)	-0,703	0,863	76
couple kiss[bw]	ph_031	59,01 (27,59)	-0,549	0,546	75	16,64 (35,99)	-0,39	-0,028	75
couple erotic[bw]	ph_032	48,69 (31,09)	-0,233	-0,37	75	20,48 (36,92)	-0,404	0,482	75
couple kiss	ph_033	57,27 (29,82)	-0,554	0,383	75	23,85 (37,76)	-0,457	0,258	75
couple bed	ph_034	56,92 (27,98)	-0,341	-0,499	75	7,15 (40,52)	-0,801	0,103	75
female couple	ph_035	31,25 (35,45)	0,085	0,21	75	2,36 (40,22)	-0,372	0,562	75
couple bed	ph_036	63,28 (26,74)	-0,219	-0,728	75	9,67 (38,34)	-0,549	0,715	75
couple kiss[bw]	ph_037	57,07 (34,21)	-1,052	1,973	75	4,69 (37,90)	-0,361	0,267	75
couple kiss	ph_038	51,49 (28,85)	-0,476	0,77	75	27,43 (34,91)	-0,382	0,706	75
female erotic	ph_039	35,01 (36,27)	-0,136	-0,335	75	21,27 (40,75)	-0,672	0,909	75
couple kiss	ph_040	59,32 (26,69)	-0,413	0,001	75	18,33 (39,49)	-0,653	0,51	75
couple kiss	ph_041	62,56 (25,70)	-0,394	-0,187	75	-1,15 (45,27)	-0,018	-0,409	75
couple erotic[bw]	ph_042	58,17 (26,93)	-0,017	-0,903	75	34,95 (30,31)	-0,053	0,016	75
couple erotic	ph_043	50,87 (30,43)	-0,078	-0,824	75	34,05 (33,70)	-0,289	0,308	75
couple kiss	ph_044	63,40 (26,58)	-0,7	0,661	75	19,52 (44,24)	-0,476	-0,573	75
couple erotic	ph_045	56,93 (28,88)	-0,453	0,155	75	35,72 (33,42)	-0,819	1,522	75

Description	Picnum	Valence-Rating				Arousal-Rating			
		M (SD)	skew	kurt	N	M (SD)	skew	kurt	N
couple kiss	ph_046	49,34 (27,70)	0,179	-0,826	74	25,65 (34,19)	-1,192	2,952	74
couple lust	ph_047	43,69 (28,23)	0,459	-0,545	74	-8,16 (40,03)	-0,104	-0,289	74
couple lust	ph_048	57,00 (27,48)	0,141	-1,151	74	23,76 (36,68)	-0,674	1,022	74
couple erotic[bw]	ph_049	53,68 (27,74)	-0,059	-0,707	74	34,55 (35,06)	-1,167	1,787	74
couple happy	ph_050	56,07 (22,64)	0,344	-0,502	74	15,80 (40,27)	-0,619	-0,036	74
couple lust	ph_051	50,14 (27,35)	0,327	-0,791	74	19,51 (37,18)	-1,132	1,424	74
couple kiss	ph_052	52,62 (29,28)	-0,152	-0,438	74	31,80 (34,32)	-1,092	2,231	74
couple erotic	ph_053	58,80 (28,87)	-0,199	-0,966	74	32,55 (37,48)	-1,174	2,102	74
couple lust	ph_054	56,46 (26,29)	0,21	-1,034	74	23,42 (40,84)	-1,119	1,53	74
couple lust	ph_055	39,77 (32,03)	0,196	-0,45	74	6,82 (37,44)	-0,492	0,516	74

female kiss	ph_056	46,19 (31,02)	-0,238	0,275	74	19,65 (39,43)	-0,135	-0,157	74
couple erotic	ph_057	52,96 (29,74)	0,076	-1,008	74	33,78 (39,09)	-0,735	0,571	74
couple lust	ph_058	48,53 (33,47)	-0,288	-0,364	74	32,08 (35,96)	-1,005	2,664	74
couple erotic[bw]	ph_059	54,85 (32,07)	-0,78	2,227	74	33,51 (39,95)	-1,073	1,403	74
couple lust	ph_060	42,07 (34,41)	-0,193	-0,03	74	29,08 (39,26)	-0,626	1,035	74

Note. M = Mean, SD = Standard Deviation, skew = skewness, kurt = kurtosis, N = number of participants rating each picture, ph = positive/high arousal. Picture sets were defined as follows: Set1 = Picnum 001-015; Set2 = Picnum 016-030; Set3 = Picnum 031-045; Set 4 = Picnum 046-060.

Table S3

Description and statistical values of the negative low arousing (nl) pictures.

Description	Picnum	Valence-Rating				Arousal-Rating			
		M (SD)	skew	kurt	N	M (SD)	skew	kurt	N
females cry	nl_001	-49,43 (32,04)	0,52	0,636	77	11,94 (33,71)	-0,756	-0,111	77
person alone	nl_002	-64,94 (25,00)	0,483	-0,264	77	12,40 (41,42)	-0,369	-0,027	77
child cry[bw]	nl_003	-45,92 (30,09)	1,207	6,124	77	17,45 (27,76)	-1,308	3,16	77
child cry[bw]	nl_004	-48,18 (34,92)	0,77	1,759	77	22,12 (34,75)	-0,327	0,665	77
family homeless	nl_005	-34,43 (46,30)	0,691	0,151	77	-,29 (43,98)	-0,4	-0,596	77
family cry	nl_006	-45,32 (31,83)	0,439	0,368	77	15,84 (35,48)	-0,834	0,752	77
child weapon	nl_007	-81,95 (25,12)	2,01	4,763	77	47,57 (38,52)	-0,484	-0,345	77
family homeless	nl_008	-68,82 (29,37)	1,289	2,047	77	26,57 (40,39)	-0,795	0,49	77
homeless	nl_009	-67,70 (24,60)	0,506	-0,262	77	16,94 (36,79)	-0,428	0,676	77
child sad	nl_010	-53,77 (26,42)	0,12	-0,73	77	8,66 (40,26)	-0,471	-0,275	77
homeless drunk	nl_011	-58,18 (28,66)	0,651	1,312	77	-4,16 (36,88)	0,14	0,198	77
child abuse	nl_012	-63,01 (27,72)	0,322	-0,551	77	26,14 (33,24)	-0,767	1,21	77
adult drunk	nl_013	-54,83 (30,75)	0,213	-0,181	77	-5,10 (41,31)	0,114	0,224	77
car theft	nl_014	-60,45 (31,38)	0,569	0,006	77	23,81 (38,92)	-0,691	1,1	77
child cry	nl_015	-52,09 (28,54)	-0,17	-1,075	77	14,95 (36,90)	-0,675	0,946	77
child abuse	nl_016	-75,96 (23,64)	1,195	0,993	76	33,55 (40,68)	-0,672	0,288	76
children cry	nl_017	-65,22 (25,74)	0,932	1,484	76	13,54 (45,18)	-0,318	-0,433	76
old person sad[bw]	nl_018	-46,83 (29,26)	0,002	-0,321	76	5,45 (41,87)	-0,566	-0,708	76
child abuse	nl_019	-55,14 (31,62)	1,4	6,166	76	12,72 (36,81)	-0,627	0,081	76
children fear	nl_020	-62,83 (24,42)	0,468	-0,173	76	32,84 (33,47)	-1,093	1,581	76
child sad	nl_021	-43,54 (22,84)	-0,431	0,11	76	9,25 (33,96)	-0,62	0,186	76
people sad	nl_022	-65,84 (22,76)	0,118	-1,071	76	23,41 (33,32)	-0,881	0,826	76
child sad	nl_023	-42,68 (26,60)	0,041	-0,08	76	-10,76 (38,88)	0,155	-0,797	76
nuclear explosion	nl_024	-60,57 (38,65)	1,078	1,028	76	38,86 (44,36)	-1,041	0,856	76

child sad[bw]	nl_025	-55,86 (27,10)	-0,06	-1,012	76	15,47 (34,70)	-0,61	-0,567	76
child sad[bw]	nl_026	-53,95 (24,58)	-0,087	-0,69	76	22,32 (34,64)	-0,209	-0,047	76
child sad[bw]	nl_027	-18,30 (36,73)	0,517	1,264	76	-27,96 (38,27)	0,379	0,172	76
female sad[bw]	nl_028	-18,80 (36,50)	0,383	0,473	76	-16,93 (31,15)	-0,012	-0,156	76
child tears	nl_029	-59,17 (25,46)	-0,07	-1,112	76	20,41 (40,17)	-0,557	0,183	76
family sad	nl_030	-58,04 (28,69)	0,954	1,96	76	25,34 (34,45)	-0,516	0,219	76
female sad	nl_031	-46,48 (31,17)	0,142	-0,194	75	-1,68 (34,05)	-0,38	-0,617	75
homeless	nl_032	-53,57 (26,30)	0,074	-0,719	75	3,67 (33,63)	-0,404	-0,384	75
child sad	nl_033	-71,85 (27,80)	0,971	0,8	75	32,23 (39,64)	-0,511	0,142	75
adult drunk	nl_034	-49,95 (31,23)	0,574	0,796	75	2,68 (40,69)	-0,236	0,226	75
adult sad	nl_035	-54,47 (25,61)	-0,162	-0,837	75	15,53 (28,72)	-0,536	-0,338	75
social exclusion	nl_036	-59,01 (27,14)	0,07	-1,265	75	7,95 (32,74)	-0,742	1,313	75
child sad	nl_037	-54,49 (26,03)	-0,176	-0,871	75	13,77 (36,70)	-0,598	0,344	75
poverty	nl_038	-43,43 (26,10)	-0,202	-0,423	75	8,28 (34,19)	-0,823	0,791	75
mold	nl_039	-54,92 (33,28)	0,413	-0,841	75	12,49 (50,37)	-0,67	-0,273	75
female sad	nl_040	-54,44 (24,06)	-0,326	-0,986	75	5,67 (34,36)	-0,452	0,757	75
cigarette buds	nl_041	-58,47 (32,35)	0,415	-0,825	75	18,41 (41,98)	-0,36	0,309	75
poverty	nl_042	-55,55 (26,85)	-0,217	-1,012	75	5,83 (36,19)	-0,474	0,511	75
child sad	nl_043	-56,20 (25,90)	-0,129	-1,126	75	-3,05 (33,47)	-0,382	-0,475	75
adult sad	nl_044	-55,73 (28,97)	0,039	-0,703	75	5,19 (35,66)	-0,476	0,404	75
female sad	nl_045	-52,63 (28,43)	0,181	-0,401	75	1,29 (34,07)	-0,573	1,258	75

Description	Picnum	Valence-Rating				Arousal-Rating			
		M (SD)	skew	kurt	N	M (SD)	skew	kurt	N
vomit	nl_046	-55,03 (31,24)	0,078	-1,111	74	22,72 (36,48)	-0,101	0,542	74
female tears	nl_047	-46,76 (32,11)	0,381	0,384	74	9,89 (28,54)	-0,098	0,347	74
female sad	nl_048	-46,42 (25,15)	-0,17	-0,489	74	-1,09 (36,63)	-0,161	0,327	74
child sad	nl_049	-41,24 (28,15)	0,629	1,263	74	19,50 (30,90)	-0,194	0,84	74
garbage	nl_050	-59,96 (27,91)	0,504	-0,561	74	16,49 (39,37)	-0,267	-0,06	74
female sad	nl_051	-44,38 (24,42)	-0,567	-0,244	74	-14,47 (28,57)	0,371	0,3	74
pollution	nl_052	-76,00 (23,33)	1,069	0,817	74	28,99 (40,68)	-0,541	0,155	74
female tears	nl_053	-54,66 (25,38)	0,105	-0,773	74	10,43 (29,58)	-0,374	0,505	74
child tears	nl_054	-50,20 (25,38)	-0,03	-0,323	74	13,11 (28,24)	0,079	1,197	74
social exclusion	nl_055	-43,39 (31,80)	0,562	0,732	74	-,32 (30,42)	-0,779	0,422	74

child drunk	nl_056	-63,49 (27,65)	0,788	0,676	74	17,12 (40,62)	-0,345	-0,005	74
child tears	nl_057	-46,53 (28,57)	0,809	3,634	74	11,99 (33,48)	-0,067	-0,217	74
female sad	nl_058	-36,15 (28,51)	-0,025	-0,09	74	-17,43 (33,22)	-0,208	0,042	74
destruction	nl_059	-78,01 (20,32)	0,7	-0,595	74	33,05 (36,27)	-0,309	-0,209	74
child sad	nl_060	-41,58 (30,78)	0,273	0,354	74	1,51 (35,88)	-0,007	-0,136	74

Note. M = Mean, SD = Standard Deviation, skew = skewness, kurt = kurtosis, N = number of participants rating each picture, nl = negative/low arousal. Picture sets were defined as follows: Set1 = Picnum 001-015; Set2 = Picnum 016-030; Set3 = Picnum 031-045; Set 4 = Picnum 046-060.

Table S4

Description and statistical values of the negative high arousing (nh) pictures.

Description	Picnum	Valence-Rating				Arousal-Rating			
		M (SD)	skew	kurt	N	M (SD)	skew	kurt	N
family cry	nh_001	-70,86 (27,44)	1,093	1,082	77	30,36 (38,78)	-0,966	1,027	77
teenager violence	nh_002	-69,42 (25,48)	0,574	-0,149	77	45,48 (38,20)	-0,846	0,894	77
child disease	nh_003	-83,95 (21,65)	1,769	3,676	77	47,81 (46,65)	-1,309	1,528	77
gun	nh_004	-71,01 (35,86)	2,503	8,597	77	51,56 (39,32)	-1,246	2,477	77
old female cry	nh_005	-70,61 (25,48)	0,876	0,357	77	27,71 (34,96)	-0,906	2,062	77
dog attack	nh_006	-51,66 (31,76)	0,306	-0,624	77	42,65 (35,07)	-0,868	1,215	77
child abuse	nh_007	-55,90 (31,83)	0,378	-0,402	77	30,38 (38,68)	-1,125	2,005	77
couple abuse	nh_008	-40,29 (45,01)	0,712	0,398	77	16,52 (46,89)	-0,713	0,03	77
people fear	nh_009	-68,83 (31,35)	1,122	1,098	77	37,70 (36,78)	-0,906	1,093	77
war victims	nh_010	-82,99 (23,85)	2,373	7,493	77	49,09 (41,23)	-1,842	4,424	77
couple abuse	nh_011	-71,64 (29,55)	2,472	11,793	77	43,78 (35,06)	-0,956	1,109	77
drug overdose	nh_012	-67,90 (34,30)	1,418	2,359	77	28,34 (43,19)	-0,777	0,563	77
adult violence[bw]	nh_013	-71,04 (27,33)	0,72	-0,129	77	38,90 (34,87)	-0,925	0,895	77
child abuse	nh_014	-66,65 (31,02)	1,066	1,047	77	43,32 (36,86)	-0,586	0,21	77
violence weapon	nh_015	-68,51 (29,49)	1,043	1,124	77	39,92 (37,03)	-0,772	0,561	77
garbage pollution	nh_016	-71,82 (22,50)	0,367	-1,056	76	26,83 (40,42)	-0,85	0,249	76
female cry/rage	nh_017	-60,63 (29,57)	1,137	1,541	76	39,53 (30,73)	-0,467	0,733	76
bird oil pollution	nh_018	-73,84 (32,98)	1,785	3,923	76	30,67 (46,82)	-0,854	0,398	76
war victim	nh_019	-71,21 (30,11)	1,901	4,843	76	48,61 (34,50)	-1,574	3,664	76
feces toilet	nh_020	-78,87 (26,33)	1,428	1,58	76	54,13 (32,30)	-0,564	-0,266	76
gun	nh_021	-71,25 (26,41)	0,573	-0,646	76	51,86 (45,83)	-1,408	1,572	76
war victim	nh_022	-76,51 (24,70)	1,039	0,369	76	49,36 (30,87)	-1,435	3,334	76
dentist	nh_023	-30,43 (33,79)	-0,239	-0,414	76	14,95 (48,12)	-0,353	-0,477	76
drug abuse	nh_024	-77,74 (21,04)	0,873	-0,054	76	44,49 (32,69)	-0,471	0,646	76

disease	nh_025	-77,86 (27,70)	1,543	2,671	76	52,55 (33,52)	-0,945	1,598	76
child abuse	nh_026	-85,70 (19,88)	1,715	2,155	76	60,87 (29,31)	-1,091	2,843	76
malnourishment	nh_027	-79,89 (30,70)	3,059	13,057	76	47,76 (37,25)	-0,77	0,068	76
anorexia[bw]	nh_028	-67,24 (37,65)	2,026	4,925	76	36,21 (39,30)	-0,551	0,16	76
adult violence	nh_029	-71,38 (25,00)	0,982	0,516	76	50,80 (26,70)	-0,385	0,311	76
drug abuse	nh_030	-73,25 (22,90)	0,757	0,131	76	33,17 (41,06)	-0,85	1,106	76
adult violence	nh_031	-79,29 (21,23)	0,915	-0,033	75	53,27 (31,14)	-0,573	0,093	75
destruction	nh_032	-69,87 (26,29)	0,986	1,251	75	35,05 (30,70)	-0,45	0,388	75
decaying teeth	nh_033	-78,16 (24,79)	1,248	0,774	75	60,51 (29,08)	-0,713	0,07	75
people cry	nh_034	-47,01 (45,38)	1,206	1,194	75	28,97 (27,01)	-0,266	0,463	75
adult violence	nh_035	-81,95 (20,27)	1,021	0,041	75	58,93 (30,26)	-0,793	0,881	75
couple abuse	nh_036	-69,89 (29,42)	0,999	0,627	75	45,37 (32,27)	-0,636	0,972	75
car crash	nh_037	-70,52 (29,82)	1,469	3,148	75	46,09 (30,07)	-0,564	1,198	75
disaster victims	nh_038	-64,52 (37,26)	1,734	3,347	75	47,27 (30,23)	-0,734	0,77	75
people cry	nh_039	-64,45 (29,98)	1,279	2,859	75	29,92 (33,04)	-0,9	1,353	75
destruction	nh_040	-71,84 (22,03)	0,293	-1,035	75	36,97 (29,02)	0,121	0,017	75
gun	nh_041	-67,52 (29,94)	0,716	-0,609	75	47,03 (40,36)	-1,296	2,497	75
couple abuse	nh_042	-78,12 (24,25)	1,224	0,846	75	51,04 (30,10)	-0,614	1,125	75
couple violence	nh_043	-70,51 (27,21)	0,631	-0,79	75	40,04 (34,32)	-0,399	-0,072	75
war victims	nh_044	-81,17 (24,66)	2,196	7,195	75	56,08 (34,82)	-1,077	2,471	75
child cry	nh_045	-66,67 (34,34)	1,89	6,137	75	39,33 (33,26)	-0,988	2,498	75

Description	Picnum	Valence-Rating				Arousal-Rating			
		M (SD)	skew	kurt	N	M (SD)	skew	kurt	N
adult violence	nh_046	-45,32 (30,55)	-0,128	-0,92	74	24,49 (36,00)	-0,802	0,782	74
child abuse	nh_047	-69,64 (23,80)	0,25	-1,13	74	45,76 (29,83)	-0,486	0,316	74
teenager violence	nh_048	-59,35 (29,17)	0,627	1,132	74	36,86 (29,17)	-0,457	1,137	74
car crash	nh_049	-79,19 (21,56)	1,072	0,479	74	45,23 (35,15)	-0,501	0,362	74
child abuse	nh_050	-65,88 (28,42)	0,765	0,265	74	39,58 (32,30)	-0,108	-0,347	74
child sad/rage	nh_051	-44,11 (22,11)	-0,052	0,487	74	15,16 (35,76)	-0,687	0,951	74
teenager violence	nh_052	-76,30 (23,02)	0,727	-0,563	74	52,97 (29,61)	-0,142	-0,664	74
car crash	nh_053	-77,73 (21,90)	0,665	-0,65	74	50,07 (33,37)	-0,368	-0,403	74
car crash	nh_054	-78,74 (20,29)	0,634	-0,782	74	46,30 (32,42)	-0,463	0,504	74

child violence	nh_055	-65,82 (25,16)	0,375	-0,693	74	41,51 (32,33)	-0,486	0,865	74
adult violence	nh_056	-69,78 (28,57)	1,044	0,645	74	44,03 (37,77)	-0,516	0,189	74
child rage	nh_057	-36,76 (31,36)	0,473	0,491	74	20,58 (35,12)	-0,582	0,853	74
drug abuse	nh_058	-72,16 (26,41)	0,752	-0,325	74	39,23 (36,78)	-0,501	0,579	74
couple abuse[bw]	nh_059	-75,00 (25,57)	1,073	0,399	74	47,64 (30,71)	-0,491	0,214	74
drug overdose[bw]	nh_060	-67,50 (28,36)	0,585	-0,639	74	34,95 (37,61)	-0,393	-0,107	74

Note. M = Mean, SD = Standard Deviation, skew = skewness, kurt = kurtosis, N = number of participants rating each picture, nh = negative/high arousal. Picture sets were defined as follows: Set1 = Picnum 001-015; Set2 = Picnum 016-030; Set3 = Picnum 031-045; Set 4 = Picnum 046-060.
